# SAFER-Dem: generating co-designed adaptations to a discharge care planning bundle for people living with dementia

**DOI:** 10.1136/bmjopen-2025-109677

**Published:** 2026-03-26

**Authors:** Natasha Tyler, Grace Harbinson, Sally Jane Giles, Oládayò Bífárìn, Sahdia Parveen, Catherine Robinson, Maria Panagioti

**Affiliations:** 1 NIHR School for Primary Care Research, NIHR Greater Manchester Patient Safety Research Collaboration, Division of Population Health, Health Services Research and Primary Care, The University of Manchester, Manchester, UK; 2 Psychology Department, University of York, York, UK; 3 Division of Population Health, Health Services Research and Primary Care, The University of Manchester Faculty of Medical and Human Sciences, Manchester, UK; 4 Mersey Care NHS Foundation Trust, Liverpool, UK; 5 School of Nursing and Advanced Practice, Liverpool John Moores University, Liverpool, UK; 6 National Institute for Health and Care Research (NIHR), London, UK; 7 Centre for Applied Dementia Studies, Faculty of Health, University of Bradford, Bradford, UK; 8 Division of Nursing, Midwifery & Social Work School of Health Sciences, Faculty of Biology, Medicine and Health, The University of Manchester, Manchester, UK

**Keywords:** PSYCHIATRY, MENTAL HEALTH, Health Services

## Abstract

**Abstract:**

**Objectives:**

To gather insights from people living with dementia, unpaid carers and professionals to co-design and refine a dementia-inclusive adaptation of the SAFER-Mental Health discharge care bundle, SAFER-Dem, that addresses challenges in the discharge process from mental health inpatient settings. A secondary objective was to assess how stakeholders interact with and understand the revised materials, focusing on usability and the perceived effectiveness of the dementia-inclusive adaptations.

**Design:**

Qualitative co-design study involving sequential workshops and ‘think-aloud’ usability interviews, guided by participatory design and cognitive interviewing methods.

**Setting:**

Mental health inpatient settings in the UK.

**Participants:**

17 stakeholders (nine people with lived experience as a person living with dementia or carer and eight healthcare professionals) took part in four co-design workshops. 12 additional stakeholders (four people living with dementia, four unpaid carers and four professionals) participated in ‘think-aloud’ interviews. Participants were recruited via NHS Trust networks, advisory groups and social media.

**Results:**

Participants highlighted widespread dissatisfaction with current discharge procedures, especially communication failures and environmental barriers. Changes to SAFER-Dem included simplified materials, flexible timing of delivery, realistic imagery and additional aids such as scenario cards and talking mats. Three main usability themes emerged: appropriateness, practical changes and usability.

**Conclusions:**

The SAFER-Dem care bundle was well received by stakeholders, showing promise for improving discharge quality for people living with dementia. Participants identified areas for improvement to enhance accessibility and effectiveness. Findings suggest that with further evaluation, SAFER-Dem could become a valuable tool in supporting dementia-inclusive discharge practices. Research and co-design with people living with dementia have been instrumental in understanding experiences during discharge.

**STRENGTHS AND LIMITATIONS OF THIS STUDY**

- The study used rigorous participatory and ‘think-aloud’ methods, engaging people living with dementia, unpaid carers and professionals throughout all stages of design and evaluation.
- A lived experience advisory group contributed to shaping workshop design, recruitment and interpretation, enhancing credibility and co-production integrity.
- Online recruitment and consent procedures may have inadvertently excluded individuals with more severe dementia or limited digital literacy.
- The sample size limits generalisability; future work should test SAFER-Dem with more diverse populations and in real-world clinical implementation studies.

## Background

 The WHO recognises dementia as a global public health problem and calls for a global action plan.^1^ Dementia can profoundly affect quality of life.^1^ Historically, individuals with dementia have been excluded from research,^2^ especially from studies on transitional interventions (ie, hospital discharge to community care, generalist to specialist care).^1^ A systematic review showed many studies purposefully exclude individuals with dementia, creating gaps in understanding their needs.^3^ Another review highlighted that staff often lack clarity on the needs of people living with dementia.^4^ Research within inpatient mental health settings is scarce, potentially worsening health inequalities.^4^

Transitions between healthcare settings increase risks, such as inadequate information transfer or medication errors.^5^ People living with dementia and their unpaid carers often navigate complex, fragmented systems without adequate support.^46^,^8^ Individuals living with dementia are particularly vulnerable on discharge from mental health inpatient services, with discharge discussions occurring in only 57% of cases.^6^ This is concerning, as about one in four hospital beds is occupied by individuals with dementia.^6^ When people living with dementia or their unpaid carers are not involved in discharge discussions, it is difficult to achieve person-centred care. Barriers to effective discharge include the mental health capacity of people living with dementia and communication difficulties.^7 9^ Facilitators include social support, the ability of unpaid carers/service users to access community resources, person-centred discussions and staff working within multidisciplinary teams during discharge planning.^8^

Care bundles, like the NHS Improvement SAFER patient flow bundle,^10^ aim to improve discharge quality and safety. However, our qualitative research shows some components are less applicable in mental health settings, where admissions often occur during crises. In response, we developed the SAFER-Mental Health (SAFER-MH) care bundle,^11^ adapting existing elements and introducing new components tailored to mental health settings. The SAFER-MH care bundle facilitates patient-centred discharge discussions and addresses specific barriers and facilitators to effective discharge.^11^

People living with dementia and their unpaid carers express a desire to participate in research. To support this, innovative methods like walking interviews and graphical translations of complex forms have been employed.^2 12^ These methods could also enhance the SAFER-MH care bundle, making the patient-written discharge plan more accessible and inclusive.

### Objectives

To gather insights from people living with dementia, unpaid carers and professionals to co-design and refine a dementia-inclusive SAFER-MH care bundle that addresses challenges and gaps in the discharge processing mental health inpatient settings.

To assess how people living with dementia, unpaid carers and professionals interact with and understand the refined SAFER-MH care bundle, focusing on the usability and effectiveness of the dementia-inclusive adaptations in supporting the discharge process.

## Methods

This study involved four sequential co-design workshops and twelve ‘think-aloud’ interviews with stakeholders, including people living with dementia, unpaid carers and professionals.

### Patient and public involvement

**Figure 1:**
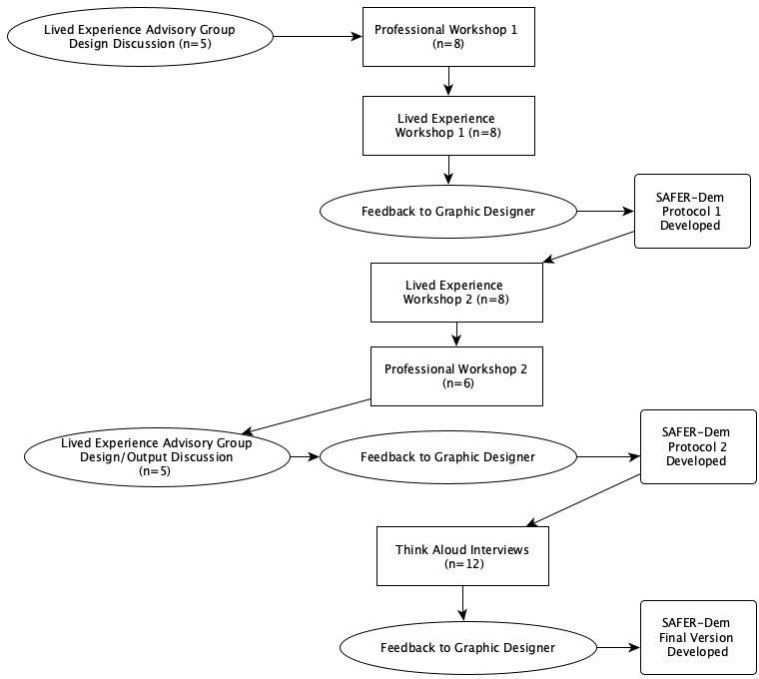
Summary of methods.

A lived experience advisory group was involved throughout the research design discussions, development and assessment of the dementia-inclusive SAFER-MH care bundle (see figure 1).

### Participants

People living with dementia, unpaid carers and professionals were involved in every stage of the co-design process. Participants were recruited through advisory groups, existing professional networks of the team and social media adverts (X, Facebook). Flyers were also shared with relevant healthcare professionals from two collaborating National Health Service (NHS) Trusts in the North of England involved in the previous SAFER-MH feasibility study.^11^ Participants were recruited a minimum of 1 week before the workshops, to provide opportunities to reflect on their involvement. Each workshop had between six and eight participants. Professional workshops included healthcare professionals, dementia design experts and academics in dementia-inclusive research. Lived experience workshops involved people living with dementia and their unpaid carers, with a graphic designer present. ‘Think-aloud interviews’ were conducted with people living with dementia, unpaid carers and healthcare professionals.

Inclusion criteria across the study were (1) individuals with lived experience of dementia; (2) healthcare professionals involved in delivering inpatient mental health services for people living with dementia and (3) unpaid carers of individuals living with dementia. Exclusion criteria were (1) individuals who are unable to read the easy access participant information sheet and summarise it back to us (a proxy measure of capacity to participate); (2) individuals who are unable to understand English; (3) children and adolescents; (4) individuals who cannot give informed consent and (5) individuals who are currently hospitalised. Dementia severity among participants with lived experience was not formally assessed; however, the capacity screening process may have indirectly excluded individuals with more severe impairment.

### Procedure

#### Co-design workshops

Workshops were held between June 2023 and October 2023. The composition and order were discussed with the project lived experience advisory group, who recommended that the professional workshop be first to provide evidence-based insights for the first lived experience workshop. The final workshop was suggested to be for professionals, serving as a final ‘sense-check’ before the ‘think-aloud’ interviews. The procedure is outlined in figure 1.

#### Workshop 1 and 2

Two sequential, but identical, workshops were conducted first with a professional stakeholder group, then repeated with a lived experience stakeholder group. The aim of the workshop was to present the original SAFER-MH care bundle and discuss adaptations to make it dementia-inclusive. The activity sequence was as follows: (1) introduction and aims; (2) summary of our work to date; (3) presentations of resources outlined in existing literature and interventions for dementia-friendly adaptations (eg, talking mats, conversation maps, pictorial representations); (4) structured discussions around overall adaptations and (5) structured discussions around adaptations to each individual component of the SAFER-MH intervention. Ideas from the first professional workshop were also shared with the lived experience group. Workshops lasted 2 hours and were facilitated by researchers with extensive experience in co-design, NT and SJG.

#### Workshop 3 and 4

Two further identical, sequential workshops were held, first with the lived experience group, and then repeated with the professional group. Similar to workshops 1 and 2, workshops 3 and 4 also lasted 2 hours and were facilitated by NT and SJG. The aim of workshops 3 and 4 was to refine the SAFER-Dem prototype 1, developed as a result of the outcomes from workshop 1 and 2. The activity schedule was as follows: (1) introductions and reiteration of the aim; (2) sharing and reviewing the developed prototype and (3) structured discussions about adaptations. Small group discussions addressed the practicalities of the changes and identified any future adjustments needed for the care bundle. Outcomes from the lived experience workshop were fed back to the professional workshop.

#### ‘Think-aloud’ interviews

Adaptations suggested in workshops 3 and 4 were incorporated into SAFER-Dem (prototype 2). 12 ‘think-aloud’ interviews were conducted, where participants read items from the SAFER-Dem care planning resources and described their thought process. One researcher (NT) led the interviews using a standardised prompt sheet to familiarise participants with the process. The prompts covered: (1) comprehension, (2) retrieval, (3) judgement and estimation and (4) formulating a response, with standardised follow-up questions (eg, whether participants could make a judgement based on retrieved information). The methodology was based on Tourangeau’s work.^13^ The interviews were conducted via MS Teams, with resources shared through screen-sharing. The sessions were audio-recorded and transcribed verbatim.

Think-aloud interviews were selected because they are grounded in cognitive psychology and usability theory, which posit that verbalising thought processes during task performance provides direct insight into users’ cognitive strategies, comprehension and interaction with an interface.^14^ This method is particularly appropriate for evaluating prototype mental health resources for people living with dementia, as it enables researchers to identify usability barriers and cognitive load in real time, ensuring that the design supports accessibility and user engagement.

### Data analysis

Demographic data of participants were collected (stakeholder group, age, gender, ethnicity). Workshop feedback from stakeholder groups was synthesised into a list of key suggested changes and provided to the graphic designer to develop protocol resources. ‘Think-aloud’ interview data underwent thematic analysis^15^ using NVivo V.12 software.^16^ A coding framework was developed inductively, whereby codes were generated directly from the data through iterative reading and re-reading of transcripts, allowing themes to emerge organically and be refined through repeated engagement with the data to ensure alignment with emergent themes and participant language. Codes were examined for similarities and differences, forming subthemes, which were then grouped into overarching topics. After initial coding, two researchers (GH and NT) met regularly to compare interpretations, review code definitions and discuss any discrepancies in coding decisions. Where differences arose, these were explored in depth by revisiting the original data and considering alternative interpretations until a shared understanding was achieved. Consensus was reached through iterative discussion rather than majority decision, ensuring that the final coding framework accurately reflected the data

## Results

### Participants

#### Workshops

In addition to the graphic designer that attended all four workshops, 17 stakeholders took part in the four workshops. This included nine people with lived experience (as a person living with dementia or a carer) and eight professionals (researchers and healthcare professionals). The ages of stakeholders ranged from 27 to 88. 12 stakeholders were White British or Scottish, three were Black British and two were (British) Pakistani. 10 stakeholders were female, seven were male (see online supplemental file 1).

#### ‘Think-aloud’ interviews

Four participants were recruited from each stakeholder group (healthcare professionals, people living with dementia and unpaid carers). Nine of the participants were female, three were male with an age range of 37 to 75. Nine participants were White British/Scottish, one mixed race, one British South Asian and one British Asian Pakistani (see online supplemental file 2).

### Workshops

In workshops 1 and 2, regarding ideas to make SAFER-MH more accessible, the groups agreed it needs to be more visual and less text heavy. However, participants preferred illustrations and graphics over cartoons (seen as patronising) and photographs (considered confusing), as a way to do this. Given the complexities of dementia, a single universal solution was deemed inadequate. Instead, a suite of resources was recommended to support varying dementia severities, including pictorially represented questions and additional tools: (1) third-person scenario cards as conversation starters and (2) ‘talking mats’ as communication aids. Key findings are summarised in online supplemental file 3. An initial prototype (SAFER-Dem prototype 1) was presented to groups in workshop 3 and 4, and groups highlighted specific adaptations to make the resources more accessible (changes to wording, images, text size, etc) to generate SAFER-Dem prototype 2.

### ‘Think-aloud’ interviews

Thematic analysis conducted using prototype 2 of SAFER-Dem revealed that responses to the ‘think-aloud’ interviews centred on two overarching topics, the first related to dissatisfaction with current discharge procedures and the second centred around practical improvement of the SAFER-Dem care bundle. The topics identified in this study represent a progression from challenges experienced with current discharge procedures to potential improvements offered by the SAFER-Dem care bundle. Dissatisfaction with discharge is driven by communication failures and environmental issues, which responses suggest could be addressed by SAFER-Dem. Further implementation considerations outlined in themes focused on appropriateness, practical changes and usability of the care bundle. Together, these interconnected topics and themes link the problems people living with dementia, unpaid carers and staff face to adaptations that can be made to refine and better implement SAFER-Dem to address these problems.

While topic 1, ‘dissatisfaction with current discharge procedures’, provides essential context by highlighting the key challenges experienced by patients and staff, it was not the primary focus of this study and therefore explored more broadly. In contrast, topic 2, ‘improvements of the SAFER-Dem care bundle’, directly addresses the research questions and objectives. As such, greater emphasis is placed on detailing and exploring the subthemes within this paper.

### Dissatisfaction with current discharge procedures

The first topic, dissatisfaction with discharge procedures, consisted of two themes: communication and environmental issues (see table 1).

**Table 1:** Summary of themes and subthemes

Theme	Subtheme
Topic 1: Dissatisfaction with current discharge procedures	
Communication	Feeling unheard
	Feeling uninformed
	Between services communication
	Lacking communication around medication
Environmental issues	Changing environments
	Acuity of wards
	Belongings going missing
Topic 2: Improvements of the SAFER-Dem care bundle	
Appropriateness	Usefulness
	Accessibility and inclusivity
	Capacity
	Idealism
Practical changes	Resources format
	Digitalisation
	Simplification and clarity
	Emotive responses
Usability	Timing
	People involved
	Additional aids

#### Communication

Communication was found to be a significant concern in four subthemes: feeling unheard, feeling uninformed, communication between services, and issues related to medication. Participants noted that individuals living with dementia sometimes feel that their unpaid carers dominate conversations in which they are capable of participating. Insufficient shared decision-making and information provision about the discharge process were prominent themes. Frustration was commonly linked to poor communication between health and social care providers, which made the process difficult to navigate, particularly around medication management.

I’ve never seen anything like this, and the times I’ve been in hospital I’ve never been given any talk through or anything.

-person with lived experience.

That’s good because they don’t tell you what you’re taking and why you’re taking it and what it does or anything, you know, it’s not very often anybody will take the time to explain to you why you’re taking this medication.

-person with lived experience.

#### Environmental issues

The environmental issues theme contained three subthemes: changing environments, acuity of wards and belongings going missing. Participants noted the challenges of navigating the transition between different environments. The busy nature of hospitals was considered overwhelming, under-resourced and understaffed, which often led to a perception that professionals lacked the capacity to engage effectively in discussions and planning. Additionally, participants expressed frustration with the issue of belongings going missing.

But most recently mother-in-law being there, trying to get hold of the discharge nurse or anyone to sit down with anyone to explain anything is nigh on impossible

-carer.

### Improvements to the SAFER-Dem care bundle

Three key themes were generated in relation to the topic improvements needed for the SAFER-Dem care bundle: (1) appropriateness, (2) practical changes and (3) usability (see table 1).

#### Appropriateness

‘Responses regarding the appropriateness of the resources focused on how participants perceived the resources and whether they would be suitable for use with individuals with dementia in their current form. Four subthemes were identified from these responses: usefulness, accessibility and inclusivity, capacity and idealism.’

#### Usefulness

Many participants noted that the resources effectively reflected the experiences of individuals with dementia. Discussions highlighted the care bundle’s value for unpaid carers, helping them assist their loved ones with answering questions and providing reassurance. Participants agreed that the care bundle would be useful for initiating and facilitating conversations and seen as a key tool for enabling individuals with dementia and their unpaid carers to contribute to shared decision-making. Utility to all stakeholders was noted.

You’re going to worry about all of that if I’m coming out of hospital. Well, where am I going to go if I’m not well enough to go home or, you know, what’s going to happen to me? …I think they’re all important, myself, yeah.

-person with lived experience.

It empowers the patient, if they’ve obviously got a level of insight into kind of knowing who is there on discharge for them for support, so, that continuity of care.

-healthcare professional.

It’s quite helpful to the family member if they’re having difficulty explaining this thing, they’ve got a prompt to help.

-Carer

#### Accessibility and inclusivity

Participants raised concerns about the accessibility of the care bundle materials, such as confusing wording or imagery that didn’t effectively convey meaning. However, most found the materials ‘fairly easy to look at’ (healthcare professional) and noted components that enhanced accessibility. Several participants highlighted the importance of considering communication differences when adapting the resources, such as creating multiple versions tailored to different accessibility needs. People living with dementia highlighted the need to change a number of images in the prototype that could be confusing.

I mean to me, it looks like a bit of cheese with a red birthday cake on top

-person with lived experience.

So, you know, is there an option of doing a large print of this as well…is there an option there of, you know, putting it in different formats, braille, et cetera?

-healthcare professional.

#### Capacity

Capacity was a recurring theme, with a general consensus that some individuals might lack the capacity to engage with the resources at all. Participants agreed that those with mild to moderate dementia could likely engage with the materials, while individuals with more severe dementia would probably need assistance from family members or unpaid carers. Contrasting views around capacity were presented, while many participants felt that it’s important to involve people in shared decision-making, some felt that in cases of greater severity, no level of adaptation would facilitate involvement.

I wouldn’t have been in the right state of mind to accept

-person with lived experience.

You know, someone with quite a good level of insight and a mild to moderate dementia might be able to answer that. But obviously, the further down the line you go in that journey, you’re not…I don’t think however dementia-friendly you make that, they’re not going to be able to understand that information

-healthcare professional.

So, these questions, I think as a carer I could comment, but for my mother if I was to ask her all these questions she wouldn’t know

-carer

#### Idealism

Some participants noted that certain elements of the resources might be unrealistic to implement due to the diverse social circumstances of individuals with dementia and constraints on professionals. Concerns were raised that, given the pressures on many wards, professionals might lack the capacity to fully engage. Similar issues were highlighted regarding some prompts, such as those related to health and nutrition.

I would be very dubious about whether or not this would actually be done honestly or whether or not it would be done because they have to do it, and whether or not it would be done at the time or from memory

-carer.

Well, they’re eating that healthy food, I mean, you know, it’s just so expensive though isn’t it

-person with lived experience.

#### Practical changes

In relation to the practical suggestions for improving the resources, four key subthemes were identified: (1) resource format, (2) digitalisation, (3) simplification and clarity and (4) emotive responses.

#### Resource format

While many of the participants felt the response options were optimal, several participants suggested alternative formats like scales or percentages for certain questions. Some also proposed enlarging response boxes to capture more meaningful discussions; however, in contrast, some participants focused on keeping response boxes small to increase brevity. For items with pre-existing options, there was support for offering a broader range of choices to accommodate diverse needs. Additionally, a few participants recommended reordering questions to enhance accessibility for individuals living with dementia.

Or if there was a percentage, like where you’ve got happy you’ve got ten per cent

- carer

Is it worthwhile having some kind of headers in there? So, like, you know, some prompts. So, like, social care, local mental health, urgent care mental health, you know, to…to, because I suppose again, that’s subjective

-healthcare professional.

I think another thing could be recreational…religion, because some dementia patients are from a religious minority.

-carer.

#### Digitalisation

Several participants highlighted the benefits of digitalising the materials, some stressed the importance of offering both digital and non-digital options to avoid excluding people who are not digitally literate. However, a number of professionals felt that with the right support, steps could be taken to support those who aren’t digitally literate. For the majority, the consensus was that individuals with dementia, especially those with mild to moderate dementia, would be receptive to a digital version of the materials, with the right support. Suggestions for digitalisation included using digital prompts for scenario cards and incorporating interactive elements.

I think, you know, I think this could even work on a laptop or computer, or iPad, you know, sitting next to the person

-carer.

Or you could have, like, an image of a suitcase with different compartments in and you drag, you know, across. What are you worried about, you know, about taking home with you? And then they drag what they feel is important

-healthcare professional.

it’s about showing someone before, informing them and obviously if they are not able to do the digital, it’s about going back to the paper copy

- healthcare professional

#### Simplification and clarity

Many participants emphasised the need for further simplification of the materials, including both images and wording, to avoid confusion. Many participants highlighted words in the protocol that could be further simplified to improve clarity. Additionally, they suggested incorporating more realistic images and examples that individuals with dementia could more easily relate to, for example, a number of people noted depicting a landline rather than mobile phone icons.

I don’t know, you could maybe find one of somebody cooking or something, you know, so then it…I mean, if you can see somebody cooking you know it’s to do with good. You know, and put healthy food around it, or somebody just maybe stood in a kitchen or something or chopping up something

-person with lived experience.

Or a landline phone. Without the hand

-person with lived experience.

I think I might be tempted to change medication to medicines instead

- carer

#### Emotive responses

Several participants noted that the materials might unintentionally provoke emotive responses and emphasised the importance of using carefully chosen language to avoid causing additional distress. Many suggestions were made on how to neutralise the language to minimise potential distress. Using the word ‘worries’ was a particular point of concern for a number of participants. Some suggested changing to a less strong word like ‘concern’ others suggested neutralising the word to ‘feelings’ to avoid causing distress. A number of participants suggested framing the questions to inspire hope or positive feelings, discussing excitement for the future rather than concerns.

But that almost feels like, oh well, I’m worried about all of those, and I wasn’t worried about them before.

-person with lived experience.

Or what are you looking forward to after leaving the hospital? That might be better

-carer.

Maybe you should change it from worry to concern

- healthcare professional

#### Usability

The final theme was the usability of the materials, focusing on their procedural application. Three subthemes emerged in these responses: timing, people involved and additional aids.

#### Timing

Participants discussed the optimal timing for introducing the resources, noting that providing it within the first 48 hours, as suggested in the existing SAFER-MH guidance, might cause confusion. There was general consensus that the timing should be tailored to each individual, based on when they are ready to engage. Discussions also touched on the form’s length and the time required to complete it. While the overall length was deemed acceptable, some participants noted that individuals might benefit from completing it in smaller sessions.

Doing it right at the beginning of somebody’s admission I think would be confusing for most people.

-carer.

Case-by-case, if a person’s been section, not ask yet

- Person living with dementia

a doctor/nurse who understands this sort of thing, that can actually hopefully couch it in a way that they can then keep drip feeding them what it’s going to be all about

- carer

#### People involved

Discussions also centred on who should implement the intervention. Many healthcare professionals suggested that healthcare assistants could deliver the materials, with support from a qualified clinician if needed. Contrastingly, some of the lived experience participants showed preference for a specialist delivery. Participants also debated whether the individual with dementia would benefit from having a carer present. Views were divergent across participants; while some felt carer involvement was essential, others were concerned it might overshadow the person’s voice and undermine their capabilities. This view was most commonly expressed by people living with dementia, some of whom expressed a preference to use the materials independently at first, without carer support.

I think, if at all possible, from my experience, it is best for the patient to do it without a carer there and then bring a carer in afterwards

-person with lived experience.

Some of these important discussions can then take place between the family member and the ward team

- carer

I think it could be done by, yeah, an HCA. I suppose they would just need that training about prompting. You know, it’s not just a tick box, it’s a discussion aid, and so long as they understand that it was a discussion aid, then I don’t necessarily think it needs to be a qualified nurse.

--healthcare professional.

A dementia specialist within the hospital needs to have that sort of discussion with them

-carer

#### Additional aids

Some participants highlighted the benefits of incorporating additional components into the intervention, such as memory aids to support individuals with dementia during and after the completion of the materials. Examples included audio aids and a contact card. However, the majority of participants did not propose any additional aids.

I’m not sure if you could use a cassette player, because some patients are used to listening to a lot of audio tapes and they can recall their memory by listening to audio tapes

-carer

A key contact card. But then you could have on the other side of it the crisis plan

- healthcare professional

#### Output of ‘think-aloud’ Interviews

**Figure 2:**
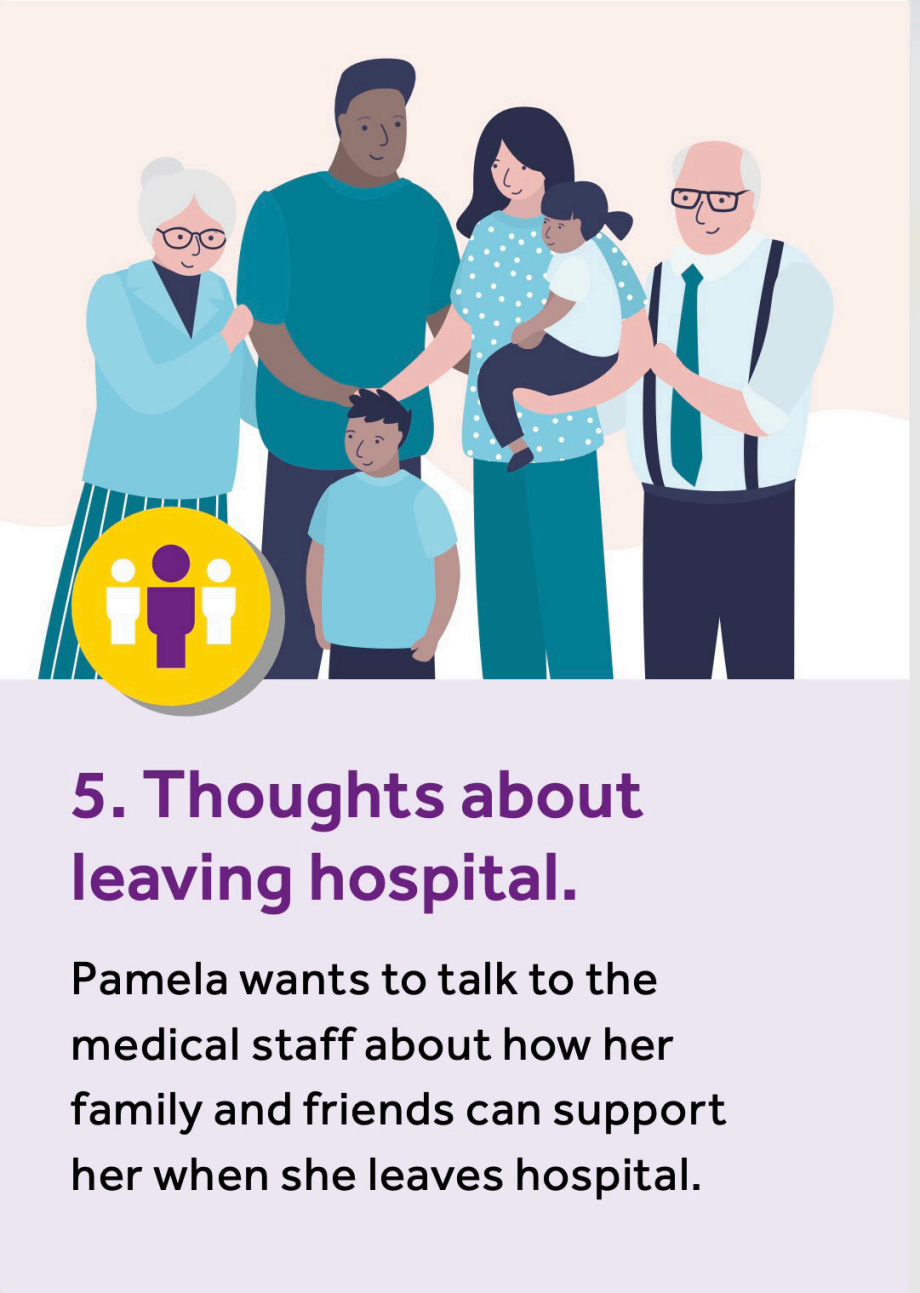
Example scenario card.

The data from the ‘think-aloud’ interviews informed another phase of adaptation whereby a final prototype for this stage of the co-design process was developed. Although we expect wider feasibility testing will be needed before implementation. Figures2  3 provide examples of the resources developed at the end of the process.

**Figure 3:**
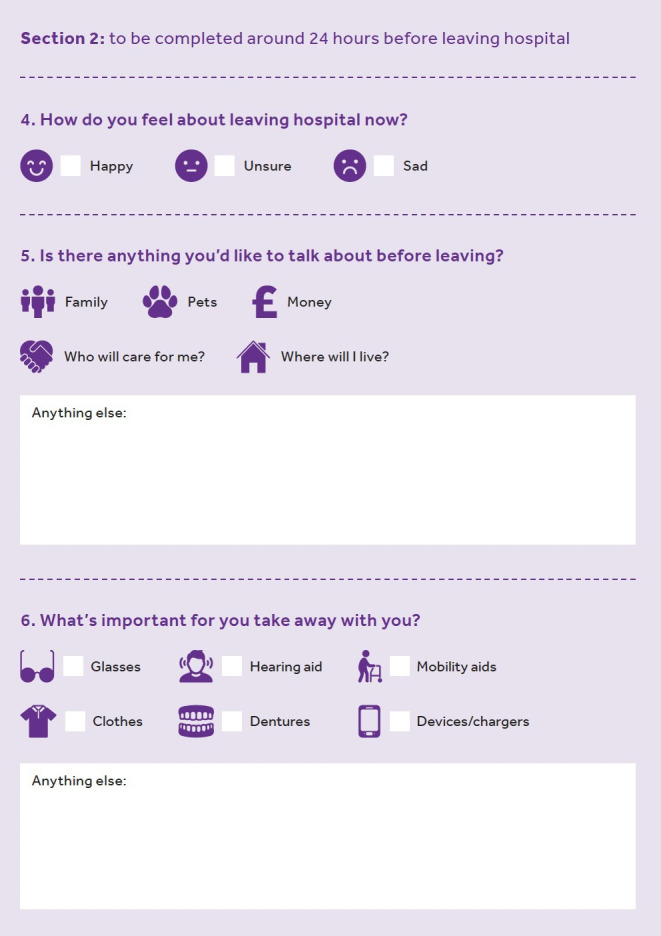
Example page of discharge planning resources.

## Discussion

This study co-designed a dementia-inclusive version of the SAFER-MH care bundle with people living with dementia, unpaid carers and professionals. The SAFER-Dem bundle was well received and the participants highlighted key improvements for better accessibility and dementia inclusivity. Key recommendations that were incorporated included simplifying materials and creating additional resources to support dementia severities. Participants preferred realistic illustrations over cartoons and photographs. Effective implementation was also emphasised, including flexible timing, engaging carers based on needs and incorporating memory support tools. These findings highlight the need for material refinements and careful implementation to support people living with dementia, their unpaid carers and healthcare professionals.

The co-design process highlighted ongoing issues in discharge procedures, particularly around communication and environment, areas directly addressed by the SAFER-Dem intervention. These findings align with existing literature, which points to healthcare professionals’ limited understanding of dementia-related needs. Participants often felt unheard and uninformed. Many individuals with dementia and their unpaid carers had never encountered a tool like SAFER-Dem and were unfamiliar with shared decision-making in hospital discharge. Some questioned whether people living with dementia are genuinely included in these discussions or if decisions should be left to professionals, reflecting research showing that only 57% of discharge discussions involve the person with dementia.^6^ In the UK, the Mental Capacity Act 2005^17^ requires that capacity is assumed unless proven otherwise and that all practicable steps are taken to support decision-making. SAFER-Dem operationalises this principle by providing simplified materials and facilitative resources. Additionally, our study corroborates literature highlighting the increased risk and vulnerability associated with transitions between health and care services,^5^ with participants reporting similar challenges navigating a fragmented system.

Previous research has identified both facilitators and barriers to effective discharge, and SAFER-Dem aims to leverage these facilitators to address existing barriers. Facilitators such as social support and the ability to use community resources are critical for effective discharge.^8^ Participants highlighted the importance of social support, stressing that while SAFER-Dem helps unpaid carers ask relevant questions, it should not overshadow the voice of the person with dementia. They viewed SAFER-Dem as a valuable tool for unpaid carers, assisting them in navigating the discharge process. Additionally, it has the potential to improve access to community resources by encouraging discussions aimed at enhancing the well-being of individuals with dementia. This study adds to the emerging evidence base of research that aims to co-design interventions to facilitate the shared decision-making of people living with dementia and their unpaid carers,^18^ and similar barriers to engagement are present, including addressing the complexities of generalisation across dementia stages, types, severities and healthcare settings. However, the emerging evidence base suggests that co-design methodology involving people living with dementia and their unpaid carers provides a vehicle to establish novel ways to improve accessibility and break down historical barriers to involvement and shared-decision making. However, randomised controlled trials (RCTs) of co-designed interventions that facilitate shared-decision making in this population are sparse, so further data is needed to establish effectiveness.

Furthermore, there are very few RCTs of discharge interventions for people living with dementia, and those that are published tend to focus on caregiver education/involvement and/or medication management;^8^ very few involve people living with dementia in their own discharge planning. This study highlights that with the right adjustments, more people living with dementia are both interested and able to be involved in shared decision-making around discharge.

Participants also suggested incorporating additional aids and resources tailored to diverse needs to improve inclusivity. A common barrier to effective shared decision-making in discharge is the mental capacity of the service user.^7 9^ Feedback from participants highlighted the need for SAFER-Dem to be adapted to better engage individuals with severe dementia. This includes using relatable imagery and developing resources that facilitate participation even with declining capacity. As such, the ‘continuum of moments’^19^ provides a valuable lens for understanding the lived experiences of individuals with dementia by highlighting the interconnected nature of seemingly small interactions. This framework reframes moments as interconnected rather than isolated, emphasising their cumulative impact. This approach is especially relevant for addressing gaps in discharge procedures. By incorporating this perspective into discharge planning, SAFER-Dem can more effectively address the communication and environmental challenges identified in the co-design process.

### Strengths and limitations

This study is one of the few to co-design discharge planning resources that include people living with dementia in shared decision-making. Involving people living with dementia throughout the co-design process was beneficial. Participants expressed feeling unheard during existing discharge procedures and did not initially think involvement in discharge planning was possible. By the end of the co-design project, they felt optimistic that SAFER-Dem could enable this. A strength of this study is the rigorous co-design process, which allowed the resources to be developed and critically evaluated by all potential users.

However, this study has several limitations. The sample size, though comparable to similar studies,^20 21^ included only four participants in each stakeholder group, potentially missing a broader range of professional opinions and demographics which may reduce generalisability. However, usability testing literature suggests that the majority of usability problems can be identified with less than five individuals.^22^ The views of those from different varieties of socioeconomic backgrounds and ethnicities are not fully represented in this sample and further work needs to be done to ensure SAFER-DEM meets the needs of vulnerable populations.

Online recruitment may have also favoured digitally literate individuals. Despite efforts to be inclusive, the inadvertent exclusion of individuals with severe dementia, due to consent, capacity assessment and online recruitment, risks reducing the generalisability of findings and perpetuating epistemic injustice, whereby the voices of those most affected are systematically omitted from research.^23^ Future research should consider ways to promote inclusion of those with greater severity in research.

An issue raised during the ‘think-aloud’ interviews was the capacity of individuals to engage with the SAFER-Dem care bundle, highlighting a challenge in ensuring its effectiveness and accessibility for those with more profound cognitive impairments. Additionally, the intervention was refined by people living with dementia, rather than originally designed by stakeholders for those living with dementia.

### Research, policy and practice implications

Future research should test the SAFER-Dem resources with a larger, diverse group of healthcare professionals and individuals with severe dementia, as well as diverse groups (eg, ethnicity, socioeconomic status, geography). Innovative methods like walking interviews and graphical translations could improve resource usability. After validation, testing the tool’s effectiveness in improving patient outcomes and exploring implementation pathways is needed.

Implementing SAFER-Dem on inpatient mental health wards requires comprehensive staff training to build confidence in shared decision-making, communication strategies and person-centred care. Ensuring staff have the knowledge and skills to facilitate SAFER-Dem implementation will be integral. Training considerations may include protected time for staff training during work hours, ensuring training is relevant to the role and using skilled facilitators, which should be factored into implementation planning.^24^ Assessing how SAFER-Dem will be integrated into existing workflows and systems should be assessed, including digital integration, to avoid duplication and minimise disruption.^25^ Potential barriers and facilitators to implementation need further assessment. Anticipated barriers could include staff workload pressures, perceived role boundaries and uncertainty about engaging individuals with cognitive impairment in discharge planning. Anticipated facilitators could include strong leadership endorsement, clear protocols and evidence of improved patient outcomes, which could enhance staff buy-in and organisational commitment.

The study highlights key policy and practice implications. It stresses the need for greater involvement of people living with dementia and unpaid carers in discharge planning, promoting a person-centred approach. Healthcare professionals need targeted training to improve communication, shared decision-making and care plan adherence while reducing agitation and safety risks.

Additionally, dementia-inclusive resources should be prioritised, with policies supporting tools tailored to individuals with varying cognitive impairments.

### Conclusions

This study finds that the SAFER-Dem care bundle shows promise for improving discharge processes for people living with dementia. While participants recognise its value and have offered meaningful refinements during this process, it will still require further evaluation in ward contexts and additional refinement before full implementation.

SAFER-Dem could ease transitions from mental health hospitals to community settings and enhance involvement in discharge decisions. The bundle addresses communication gaps, helping people living with dementia and their unpaid carers feel heard and informed during a vulnerable time. This is crucial, given the limited involvement of individuals with dementia in decision-making. Overall, the study highlights discharge challenges and offers a novel method to improve outcomes.

## Supplementary material

10.1136/bmjopen-2025-109677 online supplemental file 1

## Data Availability

Data are available on reasonable request.
