# Supplementary material for: SAFER-Dem: generating co-designed adaptations to a discharge care planning bundle for people living with dementia
Source: BMJ Open. 2026 Mar 26;16(3):e109677. doi: 10.1136/bmjopen-2025-109677 (PMC13034322; doi:10.1136/bmjopen-2025-109677)
Supplement: online supplemental file 1 [file bmjopen-16-3-s001.docx]

**SAFER-Dem: Generating co-designed adaptations to a discharge care planning bundle for people living with dementia: Supplementary Files**

**Supplementary File 1:** : Demographic details of workshop participants. Participants marked with an asterisk (*) attended one workshop only

| Workshop Number | Experience | Gender | Age | Ethnicity |
| --- | --- | --- | --- | --- |
| Professional 1 | Researcher | F | 34 | White British |
| Professional 1 | Researcher | F | 52 | White British |
| Professional 1 and 2 | Head of Nursing | F | 38 | White British |
| Professional 1 and 2 | Ward Manager | F | 57 | White British |
| Professional 1 and 2 | Mental Health Nurse/ Researcher | M | 36 | Black British |
| Professional 1 and 2 | Researcher | F | 27 | Pakistani |
| Professional 1 and 2 | Researcher | M | 57 | White British |
| Professional 2 | *Researcher | F | 37 | British Pakistani |
| Lived Experience 1and 2 | Person living with dementia | F | 60 | White British |
| Lived Experience 1and 2 | Carer | M | 31 | Black British (African) |
| Lived Experience 1 | *Person living with dementia | F | 75 | White British |
| Lived Experience 1 and 2 | Person living with dementia | M | 65 | White Scottish |
| Lived Experience 1 and 2 | Carer | M | 33 | Black-British (Afro-Carribean) |
| Lived Experience 1 and 2 | Carer | M | 66 | White Scottish |
| Lived Experience 1 | Carer | F | 64 | White British |
| Lived Experience 1 and 2 | Carer | M | 88 | White British |
| Lived Experience 2 | *Carer | F | 40 | White British |

Supplementary file 2: Demographic table for Think Aloud Interviews

| Stakeholder group | Gender | Age | Ethnicity |
| --- | --- | --- | --- |
| Carer | F | 58 | White British |
| Carer | F | 65 | White British |
| Carer | F | 65 | Mixed Race |
| Carer | M | 39 | British South Asian |
| Healthcare Professional | F | 51 | British Asian Pakistani |
| Healthcare Professional | F | 37 | White British |
| Healthcare Professional | F | 38 | White British |
| Healthcare Professional | F | 58 | White British |
| Person living with dementia | M | 65 | White Scottish |
| Person living with dementia | M | 73 | White British |
| Person living with dementia | F | 60 | White British |
| Person living with dementia | F | 75 | White British |

Supplementary file 3: Changes made to the initial intervention to co-create the SAFER-Dem

| **Workshops 1 and 2** | | |
| --- | --- | --- |
| **Change** | **Rationale** | **Example** |
| Generate simplistic illustration style images/icons for each item on the discharge plan to help with comprehension | Providing a simplistic image next to each question can improve comprehension and prompt conversation. | ‘Key contacts’ includes a phone book icon. |
| Generate image response prompts for some questions | Providing example icons/prompts for more complex questions can help aid discussions | ‘What’s important for you to take home’ response prompts include glasses icon, dentures icon, clothes icon etc. |
| Use emojis/faces as response options to facilitate discussion in some questions | Face icons can be used provide a general overview of how the person is feeling and present an aide to guide indepth conversations. | ‘How are you feeling about leaving hospital’ present 3 smiley faces (Happy, Sad, Neutral) |
| Create Additional ‘Scenario Cards’ resource, with simple images and short descriptions to support discussions and aide memory | Providing a story of another person’s response can be used to support people who might struggle to answer a question, providing an opportunity to discuss another person’s story | ‘Healthy activities:  Steve finds going on a walk helps him to feel better.’ Accompanied by a simple illustration of a man walking. |
| **Changes that were discussed but not implemented** | | |
| To use photos to accompany questions | This could be confusing as photos would not resemble the precise object described, i.e. photos of another home or family would not be familiar to the person with dementia and could cause more confusion |  |
| To use cartoon style images to accompany questions | This was deemed too patronising |  |
| To use ‘conversation maps’ to outline patients onwards journey | This was deemed to add too much complexity |  |
| **Workshops 3 and 4** | | |
| **Change** | **Rationale** | **Example** |
| Further simplify language | Some questions are still too complex | Rather than providing a list of medications, use simple prompts (who, what, when, why) to discuss any medication concerns. |
| Scenario card prototypes discussed and ideas generated for next protocol | Ensure scenario cards are representative and inclusive, also simple to understand. |  |
| Change sizes of some images | Ensure icons are not overwhelming but big enough to be understood |  |
| Change/improve some icons | Some icons might be confusing | A pound sign ‘£’ would be better than coins |
| Provide more examples of certain things | There are multiple mobility aids provide more examples. |  |
| Make some response spaces bigger | Making some of the free text spaces bigger will allow for more meaningful conversations and documentation of responses |  |
