# Supplementary material for: SAFER-Dem: generating co-designed adaptations to a discharge care planning bundle for people living with dementia
Source: BMJ Open. 2026 Mar 26;16(3):e109677. doi: 10.1136/bmjopen-2025-109677 (PMC13034322; doi:10.1136/bmjopen-2025-109677)
Supplement: Supplementary file 2 [file bmjopen-2025-109677.reviewer_comments.pdf]

PEER REVIEW HISTORY

BMJ Open publishes all reviews undertaken for accepted manuscripts. Reviewers are asked to complete a checklist review form and are provided with free text boxes to elaborate on their assessment. These free text comments are reproduced below.

ARTICLE DETAILS

Title (Provisional)

SAFER-Dem: Generating co-designed adaptations to a discharge care planning bundle for people living with dementia

Authors

Tyler, Natasha; Harbinson, Grace; Giles, Sally Jane; Bífárìn , Oládayò; Parveen , Sahdia; Robinson, Catherine; Panagioti, Maria

---

VERSION 1 - REVIEW

Reviewer 1

Name Bray, Jennifer

Affiliation University of Worcester, The Association for Dementia Studies, St Johns Campus

Date 07-Oct-2025

COI None

I found this to be a really interesting article but think it would benefit from a thorough proofread to ensure that it is consistent and cohesive, as currently it has a slight feeling of different styles or approaches being used for different bits, and no-one has checked that it actually fits together. I've gone for minor revision as each point is very minor, but the number of comments was starting to add up.

Background, first line – should it be 'The World Health Organization recognises' instead of 'The World Health Organization's recognises'?

Background, second paragraph – it would be good to have consistency, as you mainly use 'people living with dementia' or 'people with dementia', but also use 'persons with dementia'. This sort of variation makes the article feel less cohesive, so it might be worth choosing one phrase and sticking to it.

Background, third paragraph – it should be 'in response' not 'inresponse'.

At the end of the background section you say who you are going to work with to co-design the dementia-inclusive version of SAFER-MH and will focus on addressing gaps in discharge pathways. Then, at the start of the objectives section you repeat a lot of this, but give

slightly different information (e.g. this time you mention professionals). It feels a bit repetitive, so is there a way to combine them or at least be consistent? Is the bit at the end of the background section actually needed?

Methods – I'm not sure why you are again reiterating the study aim here, especially as it's not part of the method.

It might be fine and how the journal recommends doing headings and sub-headings, but it's a bit confusing trying to work out which sections go together. At first glance the methods looks like just one very short paragraph, but I'm guessing it's actually everything up to the 'Findings' heading.

Participants – when you say that participants were recruited through 'networks' it would be useful to have more information about what you mean. Is it existing networks of project team members, local support networks, what type of networks?

Also, there is quite a lot of overlap/repetition with the Methods paragraph (e.g. four co-design workshops and twelve interviews and who they were conducted with), so is there a way to combine the two? As an aside, is it 'Think Aloud interviews' or ' 'Think Aloud Interviews' ' as you use both? Also just seen that later on you say 'think-aloud interviews' which is another variation.

Materials – does this paragraph add anything that isn't covered elsewhere? If anything, the information around the existing literature/interventions in the 'Workshop 1' section is more helpful and detailed than just saying 'resources/communication aids' here.

Co-design workshops – again, the fact that you're telling me about the four co-design workshops being held sequentially is quite repetitive and makes me think there must be a way to combine bits so that the reader is presented with all relevant information in one go.

I find it confusing that you've told the reader that there were four sequential workshops, then present them with sections headed 'Workshop 1' and 'Workshop 2', and the first thing you say is that there were two sequential workshops. It's also odd how you've just said that you did the first workshop with professionals, but in 'Workshop 1' you present them in the order of 'lived experience and professional groups'. I understand that in 'Workshop 1' you are talking about the first workshop that you held with each group, and in 'Workshop 2' you are talking about the second workshop that you held with each group, but there must be a less confusing way to describe them.

I would expect to see something around ethics or the consent process to ensure everyone had capacity to be involved in the study. If no ethics approval was required, I would also expect to see this stated. I can see that you have a statement regarding ethics approval later on, but acknowledging it within the text would be good.

Patient and public involvement – as you mention the advisory group(s) in both the 'Participants' and 'Co-design workshops' sections, it might be worth moving this section earlier so that the reader knows who they are.

Findings/participants/workshops – again the headings/sub-headings are slightly confusing. It might be worth checking the journal guidelines around numbers. Should numbers up to and including 10 be spelled out? Regardless, I find it odd that up to now you've talked about 'four workshops' and suddenly (within the same sentence even) switch to '4 workshops'. Also, when talking about the Think Aloud Interview participants you say 'three' not '3', and 'one' not '1', s why use numerals here?

Findings/workshops – is it 'SAFER-Dem' or 'SAFER-DEM'? Also, I think it might be helpful to make it clear what version of SAFER-Dem the workshop findings relate to, and which round of workshops you are referring to. For example, in the first round of workshops participants would be providing thoughts and feedback based on the existing SAFER-MH, but in the second round they would be commenting on how to make the first iteration of SAFER-Dem more accessible or dementia inclusive. At the moment, you're presenting the workshop findings together, regardless of which workshop, so it's not clear what comments relate to what. For example, when talking about a preference for illustrations, the reader doesn't know if that's because SAFER-MH has photos or cartoons which people didn't like, or if the first iteration of SAFER-Dem tried an option and people objected. Also, you say that Figures 2 and 3 provide examples of the resources developed, but at which point? After the first round of workshops, or after the second round?

Think Aloud Interviews – similar to the above, the Think Aloud interviews are providing feedback on the second iteration of SAFER-Dem, so it's probably worth making that clear.

Dissatisfaction with current discharge procedures – I assume you actually mean Table 1, and I'm not missing Table 3? It also might be worth seeing if you can find a better way to refer to the overarching 'themes' (maybe topics?) as you currently have two themes, with themes, then sub-themes (or subthemes as Table 1/3 says). It's also not that helpful when you're talking about the sub-themes and refer to other points being 'prominent themes'. I know what point you are making, but more awareness about the language would be helpful to avoid potential confusion.

Environmental issues – why are these sub-themes numbered, when the ones within 'Communication' weren't? I have no preference, but appreciate consistency. Also, in the text you say 'busyness', but in Table 1 you say 'business'.

Improvements of the SAFER-Dem care bundle – it refers to Table 4, but there is no Table 4. I assume it's the second part of Table 1 which is about 'Practical Changes to Resources' as the themes match. As an aside, the way the findings are presented makes it seem as if there are still a lot of suggestions for improvements being made, which is a bit worrying considering it's based on seeing the second iteration of SAFER-Dem, and there is no further consultation before the final version is produced. I'm not saying it's a problem, but how do you know that your final version is actually 'final'.

Appropriateness – I find it odd that you list the four sub-themes then go through them with a separate section for each, when previously the sub-themes under 'Communication' were

combined into one short paragraph, and the same was done for the sub-themes under 'Environmental issues'. I appreciate you might have more to say for some sub-themes than others, and I have no particular preference in terms of how they are presented, but again some consistency would be good. Either combine or keep each one separate, don't have a mix going on.

Practical changes – surely it should be 'four key sub-themes were identified' not 'four key themes were identified'?

General point about quotes – I'm not sure I see the benefit in having supporting quotes in Table 1 (where it's not clear if they are examples, or all of the relevant quotes) and then repeating a lot of them in the text. Do you need two quotes in the text for almost every sub-theme (or group of sub-themes)? Maybe having one key quote per sub-theme would be enough, especially if it was different from the examples given in Table 1? I appreciate not every reader will look at the quotes in Table 1, but if you've chosen key quotes to go in the text then they should really be able to get what they need from there.

Usability – to match with Table 1 and the sub-heading used in the text, I think it should be 'additional aids' not just 'aids'.

Timing – why is this quote presented in a different way to all the other quotes used?

Additional aids – I'm assuming that the second bit is a quote that hasn't been italicised.

Discussion, first sentence – why is there no mention of professionals being involved in the co-design process? I find it odd that sometimes the graphic designer is mentioned, and sometimes it's the professionals, but not both.

As before, it would be useful to know which version of SAFER-Dem comments were made about, to help provide context. Some of the wording in the first paragraph also makes it sound like further work is needed, rather than making recommendations that I assume were acted upon to create the final version.

Conclusions – as in the previous point, the wording is slightly confusing. By saying 'participants recognise its value but suggest refinements before full implementation. Once improved…' you are implying that the final version of SAFER-Dem did not address the feedback from the interviews, but I thought the whole point of the interviews was to help develop a final version.

Reviewer 2

Name Miura, Hisayuki

Affiliation National Center for Geriatrics and Gerontology, Department of Home Care and Regional Liaison Promotion

Date 08-Oct-2025

COI **None**

General comment

This study addresses an important gap in discharge planning for people living with dementia and represents a timely and valuable contribution to the field. The co-design approach and involvement of people with lived experience are commendable. However, several significant issues require attention before publication.

Comments in detail

1. Pages 6-7: In Methods

Participant selection criteria are not clearly defined, and no description of how dementia severity was assessed. Thus, the authors had better add inclusion/exclusion criteria for people with dementia (including cognitive assessment methods) and specific recruitment processes and timeline.

2. Pages 6-7: In Methods

There is insufficient detail on workshop procedures. The authors are recommended to add a detailed workshop structure (duration, activity sequence, facilitation methods). How data saturation was achieved.

3. Pages 6-7: In Methods

While the authors acknowledge "only four participants per stakeholder group," there is insufficient discussion of how this affects generalizability. The authors can provide more detailed justification for the sample size (e.g., comparison with similar studies). The authors should emphasize that the absence of people with severe dementia is a major limitation. The authors may discuss more deeply how the lack of diversity (particularly ethnic and socioeconomic) may have impacted findings.

4. Pages 6-7: Methods; In the process of thematic analyses

The coding process is not clearly described, and the theme development process is insufficiently explained. The authors may be able to add coding software used (if any), the process of developing the coding framework, and agreement rates between several researchers, or a detailed description of the discussion process.

5. Pages 6-7: In Methods; Think Aloud Interview Implementation

The authors can provide clearer theoretical justification for methodological choices.

6. Pages 6-7: In Methods; Ethical Considerations

There is insufficient detail on data protection and privacy procedures. The authors can explain the protocol for capacity assessment, the process of consent.

7. Pages 8-13: In findings

Table 1 (referenced as Tables 3 and 4 in text) is comprehensive, but the relationships between themes are unclear.

8. Pages 8-13: In findings; Quote Selection and Balance

Issues:

Some sub-themes have only 1-2 quotes, and differences in perspectives between stakeholder groups are not sufficiently explored. The authors can provide multiple perspectives for each sub-theme and include disconfirming cases or minority views.

9. Pages 13-15: In Discussion

The Discussion contextualizes findings but lacks comparison with key literature on: Other dementia-specific discharge interventions; Critical co-design methodology studies; Mental Capacity Act and its implications for practice.

10. Limited Implementation Discussion

The practical challenges of implementing SAFER-Dem are not sufficiently discussed.

The authors may be able to add to the Discussion: Staff training requirements and costs; Integration with existing workflows; Barriers and facilitators to implementation.

VERSION 1 - AUTHOR RESPONSE

Reviewer: 1

I found this to be a really interesting article but think it would benefit from a thorough proofread to ensure that it is consistent and cohesive, as currently it has a slight feeling of different styles or approaches being used for different bits, and no-one has checked that it actually fits together. I've gone for minor revision as each point is very minor, but the number of comments was starting to add up.	We thank reviewer 1 for the insightful comments and feel the manuscript has greatly improved as a result of this feedback.
Background, first line – should it be ‘The World Health Organization recognises’ instead of ‘The World Health Organization’s recognises’?	We have removed this typo (pg 3)
Background, second paragraph – it would be good to have consistency, as you mainly use ‘people living with dementia’ or ‘people with dementia’, but also use ‘persons with dementia’. This sort of	We have changed it to people living with dementia throughout thanks for highlighting this.

variation makes the article feel less cohesive, so it might be worth choosing one phrase and sticking to it.	
Background, third paragraph – it should be 'in response' not 'inresponse'.	We have changed this typo (pg 4)
At the end of the background section you say who you are going to work with to co-design the dementia-inclusive version of SAFER-MH and will focus on addressing gaps in discharge pathways. Then, at the start of the objectives section you repeat a lot of this, but give slightly different information (e.g. this time you mention professionals). It feels a bit repetitive, so is there a way to combine them or at least be consistent? Is the bit at the end of the background section actually needed?	We agree, we have deleted this final paragraph of the background (pg 4).
Methods – I'm not sure why you are again reiterating the study aim here, especially as it's not part of the method.	We have deleted the sentence about the aim, thanks for pointing this out (pg 5).
It might be fine and how the journal recommends doing headings and sub-headings, but it's a bit confusing trying to work out which sections go together. At first glance the methods looks like just one very short paragraph, but I'm guessing it's actually everything up to the 'Findings' heading.	We have capitalised the highest order sub-titles (BACKGROUND, METHODS, RESULTS) to alleviate this confusion.
Participants – when you say that participants were recruited through 'networks' it would be useful to have more information about what you mean. Is it existing networks of project team members, local support networks, what type of networks?	Thank you, we have elaborated on this to outline that it was professional networks of the team and also added the flyers shared with relevant staff in collaborating NHS Trusts.
Also, there is quite a lot of overlap/repetition with the Methods paragraph (e.g. four co-design workshops and twelve interviews and who they were conducted with), so is there a way to combine the two? As an aside, is it 'Think Aloud interviews' or ' 'Think Aloud Interviews' ' as you use both? Also just seen that later on you say 'think-aloud interviews' which is another variation.	We have removed the repetition of numbers of workshop and the word 'sequential' across the methods section to improve flow and clarity and only reference these specifics in the procedure section now.<br><br>We have standardised all references to 'Think Aloud' interviews, thanks for noting this.
Materials – does this paragraph add anything that isn't covered elsewhere? If	We have removed the materials subsection.

anything, the information around the existing literature/interventions in the 'Workshop 1' section is more helpful and detailed than just saying 'resources/communication aids' here. Co-design workshops – again, the fact that you're telling me about the four co-design workshops being held sequentially is quite repetitive and makes me think there must be a way to combine bits so that the reader is presented with all relevant information in one go.	We have removed the references to the 'four sequential workshops' throughout the methods its only now in the procedure and the initial summary paragraph, below METHODs.
I find it confusing that you've told the reader that there were four sequential workshops, then present them with sections headed 'Workshop 1' and 'Workshop 2', and the first thing you say is that there were two sequential workshops. It's also odd how you've just said that you did the first workshop with professionals, but in 'Workshop 1' you present them in the order of 'lived experience and professional groups'. I understand that in 'Workshop 1' you are talking about the first workshop that you held with each group, and in 'Workshop 2' you are talking about the second workshop that you held with each group, but there must be a less confusing way to describe them.	We have changed this to make it easier to follow and instead referenced workshops 1 and 2, then 3 and 4. We have reiterated the order of workshops explicitly to avoid confusion.
I would expect to see something around ethics or the consent process to ensure everyone had capacity to be involved in the study. If no ethics approval was required, I would also expect to see this stated. I can see that you have a statement regarding ethics approval later on, but acknowledging it within the text would be good.	We have added an ethical considerations paragraph to the methods section (pg 7).
Patient and public involvement – as you mention the advisory group(s) in both the 'Participants' and 'Co-design workshops' sections, it might be worth moving this section earlier so that the reader knows who they are.	This section has been moved to the start of the methods (pg 5).

Findings/participants/workshops – again the headings/sub-headings are slightly confusing. It might be worth checking the journal guidelines around numbers. Should numbers up to and including 10 be spelled out? Regardless, I find it odd that up to now you've talked about 'four workshops' and suddenly (within the same sentence even) switch to '4 workshops'. Also, when talking about the Think Aloud Interview participants you say 'three' not '3', and 'one' not '1', s why use numerals here?	We have capitalised the highest order subtitles to remove this confusion, in line with guidelines.<br><br>In line with BMJ Open guidelines we have spelled out all numbers below 10 throughout the manuscript.
Findings/workshops – is it 'SAFER-Dem' or 'SAFER-DEM'?<br>Also, I think it might be helpful to make it clear what version of SAFER-Dem the workshop findings relate to, and which round of workshops you are referring to. For example, in the first round of workshops participants would be providing thoughts and feedback based on the existing SAFER-MH, but in the second round they would be commenting on how to make the first iteration of SAFER-Dem more accessible or dementia inclusive. At the moment, you're presenting the workshop findings together, regardless of which workshop, so it's not clear what comments relate to what. For example, when talking about a preference for illustrations, the reader doesn't know if that's because SAFER-MH has photos or cartoons which people didn't like, or if the first iteration of SAFER-Dem tried an option and people objected. Also, you say that Figures 2 and 3 provide examples of the resources developed, but at which point? After the first round of workshops, or after the second round?	We have changed to SAFER-Dem throughout.<br><br>We have referenced the different prototypes developed during the process and described the results from each in more detail.<br><br>We have highlighted that figures 2 and 3 highlight resources created at the end of the process. We have moved the reference to figures to the think aloud section. Please see below comments to your point about the 'final' version to alleviate this confusion, that this is not a finished set of resources ready for implementation.
Think Aloud Interviews – similar to the above, the Think Aloud interviews are providing feedback on the second iteration of SAFER-Dem, so it's probably worth making that clear.	We have added this.

Dissatisfaction with current discharge procedures – I assume you actually mean Table 1, and I'm not missing Table 3? It also might be worth seeing if you can find a better way to refer to the overarching 'themes' (maybe topics?) as you currently have two themes, with themes, then sub-themes (or subthemes as Table 1/3 says). It's also not that helpful when you're talking about the sub-themes and refer to other points being 'prominent themes'. I know what point you are making, but more awareness about the language would be helpful to avoid potential confusion.	We have corrected this typo (pg 8).<br><br>We have changed reference to the overarching themes to topics as suggested.
Environmental issues – why are these sub-themes numbered, when the ones within 'Communication' weren't? I have no preference, but appreciate consistency. Also, in the text you say 'busyness', but in Table 1 you say 'business'.	We have removed the numbers from this section (pg 14). Upon reflection, we have renamed 'busyness' to 'acuity of wards'
Improvements of the SAFER-Dem care bundle – it refers to Table 4, but there is no Table 4. I assume it's the second part of Table 1 which is about 'Practical Changes to Resources' as the themes match. As an aside, the way the findings are presented makes it seem as if there are still a lot of suggestions for improvements being made, which is a bit worrying considering it's based on seeing the second iteration of SAFER-Dem, and there is no further consultation before the final version is produced. I'm not saying it's a problem, but how do you know that your final version is actually 'final'.	We have removed this typo and changed to table 1.<br><br>We have removed reference to 'final' version to avoid confusion, the 'final' version described in this paper, is the final output of this research process/paper. We however acknowledge that the resource has not been tested on wards and will need likely need further refinement after this.
Appropriateness – I find it odd that you list the four sub-themes then go through them with a separate section for each, when previously the sub-themes under 'Communication' were combined into one short paragraph, and the same was done for the sub-themes under 'Environmental issues'. I appreciate you	Exploring the first overarching theme 'Dissatisfaction with current discharge procedures' wasn't a research question or objective of this study, but these this theme emerged strongly from the inductive analysis. Therefore we chose to present it to set the context, but not describe the subthemes in detail.

might have more to say for some sub-themes than others, and I have no particular preference in terms of how they are presented, but again some consistency would be good. Either combine or keep each one separate, don't have a mix going on.	Whereas Theme 2, addresses the research objectives and we have outlined the subthemes in more detail. We have added a paragraph to the 'think aloud' interviews subsection of the results (p 10) to explain the rationale behind this decision.
Practical changes – surely it should be 'four key sub-themes were identified' not 'four key themes were identified'?	This has been changed to sub-themes, pg 11.
General point about quotes – I'm not sure I see the benefit in having supporting quotes in Table 1 (where it's not clear if they are examples, or all of the relevant quotes) and then repeating a lot of them in the text. Do you need two quotes in the text for almost every sub-theme (or group of sub-themes)? Maybe having one key quote per sub-theme would be enough, especially if it was different from the examples given in Table 1? I appreciate not every reader will look at the quotes in Table 1, but if you've chosen key quotes to go in the text then they should really be able to get what they need from there.	We have removed the quotes from table 1.
Usability – to match with Table 1 and the sub-heading used in the text, I think it should be 'additional aids' not just 'aids'.	We have amended this typo, pg 12
Timing – why is this quote presented in a different way to all the other quotes used?	We have amended the formatting of this typo in line with the rest of paper pg 12.
Additional Aids– I'm assuming that the second bit is a quote that hasn't been italicised.	We have italicised this quote, pg 13.
Discussion, first sentence – why is there no mention of professionals being involved in the co-design process?	We have added that omission, pg 13.
I find it odd that sometimes the graphic designer is mentioned, and sometimes it's the professionals, but not both.	We have removed the reference to the graphic designer in the methods, he wasn't a 'participant' as such, as he was there to understand the groups needs and implement them (pg 13).
As before, it would be useful to know which version of SAFER-Dem comments were made about, to help provide context. Some of the wording in the first paragraph also makes it sound like	We have now added a subsection to end of the results 'Output of Think Aloud Interviews' to highlight the changes that were made as a result of the 'think aloud' interviews and also to make it clear that

further work is needed, rather than making recommendations that I assume were acted upon to create the final version.	we expect further testing to be necessary before implementation. We have removed reference to 'final'.
Conclusions – as in the previous point, the wording is slightly confusing. By saying 'participants recognise its value but suggest refinements before full implementation. Once improved…' you are implying that the final version of SAFER-Dem did not address the feedback from the interviews, but I thought the whole point of the interviews was to help develop a final version.	We have removed reference to 'final' version to avoid confusion, the 'final' version described in this paper, is the final output of this research process/paper. We however acknowledge that the resource has not been tested on wards and will need likely need further refinement after this.<br><br>We have made it clearer that we acted on the suggestions, but expect further feasibility testing will be needed.

Reviewer 2

This study addresses an important gap in discharge planning for people living with dementia and represents a timely and valuable contribution to the field. The co-design approach and involvement of people with lived experience are commendable. However, several significant issues require attention before publication.	We thank reviewer 2 for the insightful comments and feel the manuscript has greatly improved as a result of this feedback.
1. Pages 6-7: In Methods<br>Participant selection criteria are not clearly defined, and no description of how dementia severity was assessed. Thus, the authors had better add inclusion/exclusion criteria for people with dementia (including cognitive assessment methods) and specific recruitment processes and timeline.	We have added the inclusion and exclusion criteria to the methods (participants section) pg 5. We have also noted how the proxy capacity measure may have indirectly excluded those with greater severity, whilst severity was not assessed.<br><br>We have added more detail around the timelines (giving one week to reflect on involvement) and greater detail about the recruitment methods.
2. Pages 6-7: In Methods<br>There is insufficient detail on workshop procedures. The authors are recommended to add a detailed workshop structure (duration, activity sequence, facilitation methods). How	We have added detail about the duration, activity and facilitation, (pg 6, methods).<br><br>We have not included reference to data saturation in the manuscript. Data saturation is a concept primarily

data saturation was achieved.	associated with qualitative methodologies such as grounded theory or thematic analysis, where the aim is to explore the breadth and depth of themes until no new insights emerge. In contrast, think-aloud usability studies are designed to identify usability issues and cognitive processes during task performance, rather than to exhaustively explore thematic content. As such, the goal was not saturation but **problem discovery and insight generation.** Usability research literature (e.g., Nielsen & Molich, 1990) consistently shows that **4–5 participants** can uncover the majority of usability problems, and additional participants offer diminishing returns. Therefore, our sample size of 12 was chosen to balance **diversity of user experience** with **practical feasibility,** and is consistent with best practices in usability testing. We used Thematic Analysis but not to explore a breadth and depth of themes in the traditional sense.
3. Pages 6-7: In Methods<br>While the authors acknowledge "only four participants per stakeholder group," there is insufficient discussion of how this affects generalizability. The authors can provide more detailed justification for the sample size (e.g., comparison with similar studies). The authors should emphasize that the absence of people with severe dementia is a major limitation. The authors may discuss more deeply how the lack of diversity (particularly ethnic and socioeconomic) may have impacted findings.	We have added references of similar think aloud studies with the same sample size and justification for the sample size (pg 16. Discussion, Limitations) . We have further discussed the ethical issues due to the inadvertent absence of people with severe dementia. We have added further discussion around diversity of the sample.
4. Pages 6-7: Methods; In the process of thematic analyses<br>The coding process is not clearly described, and the theme development process is insufficiently explained. The authors may be able to add coding software used (if any), the process of developing the coding framework, and agreement rates between several	We have added the detail around software, coding processes and a detailed description of the discussion process (pg 7).

researchers, or a detailed description of the discussion process.	
5. Pages 6-7: In Methods; Think Aloud Interview Implementation<br>The authors can provide clearer theoretical justification for methodological choices.	We have added this justification (pg 7).
6. Pages 6-7: In Methods; Ethical Considerations<br>There is insufficient detail on data protection and privacy procedures. The authors can explain the protocol for capacity assessment, the process of consent.	We have added this section into the methods, pg 7.
7. Pages 8-13: In findings<br>Table 1 (referenced as Tables 3 and 4 in text) is comprehensive, but the relationships between themes are unclear.	We have added two narrative paragraphs outlining the relationship between the themes in the 'Think Aloud' Interviews section of the results (page 10).
8. Pages 8-13: In findings; Quote Selection and Balance<br>Issues:<br>Some sub-themes have only 1-2 quotes, and differences in perspectives between stakeholder groups are not sufficiently explored. The authors can provide multiple perspectives for each sub-theme and include disconfirming cases or minority views.	As there were only 12 participants, in some subthemes there were no divergent views, as we used think aloud methodology/cognitive interviewing, comments came up as individuals worked through the resources, so we didn't seek to ask the same questions and analyse for convergence/divergence, lots of the ideas people gave were organic. We have expanded upon some divergent views and added some additional quotes where possible, we have added further detail to highlight divergences.
9. Pages 13-15: In Discussion<br>The Discussion contextualizes findings but lacks comparison with key literature on: Other dementia-specific discharge interventions; Critical co-design methodology studies; Mental Capacity Act and its implications for practice.	We have added references to other co-design studies, other discharge studies and the MCA, along with implications for practice (pg 17).
10. Limited Implementation Discussion<br>The practical challenges of implementing SAFER-Dem are not sufficiently discussed.<br>The authors may be able to add to the Discussion: Staff training requirements and costs; Integration with existing	We have added more detail to the future implementation considerations in line with comments you have made (pg 16).

workflows; Barriers and facilitators to implementation.	

VERSION 2 - REVIEW

Reviewer 1

Name Bray, Jennifer

Affiliation University of Worcester, The Association for Dementia Studies, St Johns Campus

Date 21-Nov-2025

COI

Thank you for taking onboard the previous review comments, I can see that you have made a lot of progress. However, I think there is still work to be done to improve consistency and readability, and unfortunately some of your changes have introduced new issues to resolve or made other issues more apparent.

Abstract – "Think Aloud", 'Think Aloud' and Think Aloud are all used. I know that the abstract was rewritten, so it's a shame that it was not checked. It also needs to be consistent with the rest of the article.

Abstract – what does the 'Interventions' bit add? Surely the reader has all of the information it contains by the time they reach the section.

Abstract – the 'Objectives' section indicates that you were going to co-design and redefine SAFER-MH to become SAFER-Dem, but your 'Results' and 'Conclusions' make it sound like issues were identified but changes weren't made. I think (hope) it's just a wording issue, but when you say 'Recommendations for SAFER-Dem included' (in 'Results') is that further recommendations beyond what was already done, or recommendations that were actually incorporated into SAFER-Dem during your study? Also, in 'Conclusions' is it actually that 'participants identified areas for further improvement'?

Background – it feels like the last two sentences in the first paragraph could be swapped around. That way, you talk about the systematic review, another review, then research, rather than going from the systematic review to research then back to another review. Apologies for not picking up on this last time.

Background, second paragraph – again, on reflection, I think the flow could be improved by changing the order of the sentences. I would suggest 'Transitions between…', 'People living with dementia…', Individuals living with dementia…', This is concerning…', When people living with dementia…'.

Objectives – for consistency, the second objective would benefit from using the same terminology as the first one. I.e. ‘unpaid carers’ instead of ‘carers’, and ‘the refined SAFER-MH’ rather than ‘the revised SAFER-MH’.

Methods – again, for consistency is it worth saying ‘unpaid carers’? This also applies throughout the article. Either make them carers, or unpaid carers, not a mix of both unless there is a specific reason.

Methods, Participants – I think it should be ‘of the team and social media’. It should also be ‘NHS Trusts’ to match the abstract, not ‘NHS trusts’.

Where is point 1) for the exclusion criteria?

What does PIS mean? (I know, but you haven’t said it in the text)

I realise it might be due to the journal style, but I’m still struggling with the sub-headings to work out what level things are. For example, what are the sub-sections within ‘Procedure’? I assume it’s everything up to Data Analysis, but it’s not clear. Even just putting the third level headings in italics, or inset slightly would provide some clarity.

Figure 1 – I didn’t think I could see the figure initially as it is not labelled, but I am not sure you can ‘insert’ it in two places.

Methods, Procedure, ‘Think Aloud’ Interviews – it would be good if the numbered points were in the style ‘1)’ to match other parts of the article, rather than being ‘1.’. Also, using ‘Think-aloud’ does not match the rest of the article.

Methods, Data analysis – should it be ‘emerge organically and be refined’ rather than ‘emerge organically and we refined’? The sentence ‘Two researchers...approach’ feels odd as you’ve already said about the thematic analysis and developing codes inductively, so the sentence is a bit repetitive. If you want to say who carried out the coding, you could combine it with the next sentence to be something like ‘After conducting initial coding independently, two researchers (GH, NT) met regularly…’

Methods, Ethical considerations – you need to say what GDPR stands for. Also, you’ve got a reference for the Mental Capacity Act (see your Discussion section) so you could use it here too.

Results – again, I’m struggling with the sub-headings. In this section you’ve got headings for Participants, Workshops, ‘Think Aloud’ Interviews, Workshops, ‘Think Aloud' Interviews. I assume the first set is within the ‘Participants’ section, and the first set are their own sections, but it’s not clear. Either you need to find a better way to show different levels of sub-headings, or change them to something like Workshop Participants, ‘Think Aloud' Interview Participants, Workshop Findings, and ‘Think Aloud' Interview Findings so they are self explanatory.

Results, Workshops – the sentence ‘Key workshop findings…accessibility’ doesn’t really add anything and is more of a statement. I think it could be deleted. Also, it would be good if the

numbered points were in the style ‘1)’ to match other parts of the article, rather than being ‘(1)’. If you are presenting SAFER-Dem prototype 1 (i.e. one prototype even if you had multiple copies of it), should it be ‘An initial prototype…was’ rather than ‘Initial prototypes…were’?

Results, ‘Think Aloud’ Interviews – it should be ‘and the second’ instead of 'and second’. I’m not sure the sentence ‘However, further implications…care bundle’ makes sense. Is there a word missing?

Make it clear that ‘Communication’ and ‘Environmental Issues’ are sub-headings under ‘Dissatisfaction with current discharge procedures’. I know you say in the text that they are themes within it and Table 1 reinforces it, but the reader should know from the sub-headings too.

The following are all different levels of sub-heading, but it’s difficult to tell as they all look the same - Improvements of the SAFER-Dem care bundle, Appropriateness, Usefulness. It also makes it difficult to know when all of the content for one level has been presented, and we’re now back at a higher level. It relies on the reader to keep going back to list of themes and sub-themes (or Table 1) to work out where they are, and it shouldn’t be like that. It should be clear from the text.

Consistency – are quotes in “” or ‘’ or nothing, as all are used in different places? Also, are they in italics or not italics as again there is a mix? How is the person being quoted shown, as sometimes you use — and sometimes -? Also, sometimes there is s space after it, and sometimes not, and sometimes they are in bold.

Resource format – the sentence ‘Whilst many of…certain questions’ does not make sense. In particular, it’s the bit ‘participants suggested, recommending’.

Emotive responses – saying ‘Using the word or ‘worries’’ is confusing. Have you missed out a word (i.e. using the word ‘XXX’ or ‘worries’) or should the ‘or’ not be there?

People involved – the sentence ‘Views were divergent…capabilities’ would benefit from a ; after ‘participants’. There are other sentences in the article where a ; would make them easier to read, but I’m not going to point out every instance.

Output of ‘Think Aloud’ Interviews – please do something with the first sentence as it has three part separated by commas, that don’t actually seem to belong together.

Discussion – RCT? You need to tell your readers what this stands for.

Research, Policy and Practice Implications – please revisit the sentence ‘Potential barriers…organisational commitment. It either needs a ; or to be split into two sentences.

Conclusions – the sentence ‘Participants recognise…be necessary’ needs work. I can’t quite work out what it’s trying to say. Also, is it about ‘further’ refinements being needed, or is it talking about the refinements that have already been made?

**Reviewer**	**2**
**Name**	**Miura, Hisayuki**
**Affiliation**	**National Center for Geriatrics and Gerontology, Department of Home Care and Regional Liaison Promotion**
**Date**	**26-Nov-2025**
**COI**	

This revised manuscript addresses all comments raised by Reviewers with careful and sufficient revisions, resulting in substantial improvements in coherence, methodological transparency, and clarity of reporting. I consider that it now meets the reporting standards required by BMJ Open.

VERSION 2 - AUTHOR RESPONSE

Response to Reviewers

Comment	Response
Thank you for taking onboard the previous review comments, I can see that you have made a lot of progress. However, I think there is still work to be done to improve consistency and readability, and unfortunately some of your changes have introduced new issues to resolve or made other issues more apparent.	
Abstract – "Think Aloud", 'Think Aloud' and Think Aloud are all used. I know that the abstract was rewritten, so it's a shame that it was not checked. It also needs to be consistent with the rest of the article.	Sorry about this oversight, not sure how that was missed in the original changes, now changed to 'Think Aloud' in line with full text.
Abstract – what does the 'Interventions' bit add? Surely the reader has all of the information it contains by the time they reach the section.	We agree and have deleted the interventions subsection.
Abstract – the 'Objectives' section indicates that you were going to co-design and redefine SAFER-MH to become SAFER-Dem, but your 'Results' and 'Conclusions' make it sound like issues were identified but changes weren't made. I think (hope) it's just a	I have changed 'recommendations' to 'changes to'. To indicate these changes were made.

wording issue, but when you say 'Recommendations for SAFER-Dem included' (in 'Results') is that further recommendations beyond what was already done, or recommendations that were actually incorporated into SAFER-Dem during your study?	
Also, in 'Conclusions' is it actually that 'participants identified areas for further improvement'?	I have the wording to clarify this, we now have a version that is ready for further evaluation i.e. feasibility testing (and maybe refinement based on that) so I have removed any wording that might be confusing.
Background – it feels like the last two sentences in the first paragraph could be swapped around. That way, you talk about the systematic review, another review, then research, rather than going from the systematic review to research then back to another review. Apologies for not picking up on this last time.	I have swapped the two sentences and agree this reads better.
Background, second paragraph – again, on reflection, I think the flow could be improved by changing the order of the sentences. I would suggest 'Transitions between…', 'People living with dementia…', Individuals living with dementia…', This is concerning…', When people living with dementia…'.	I have followed your guidance and changed the structure of this paragraph.
Objectives – for consistency, the second objective would benefit from using the same terminology as the first one. I.e. 'unpaid carers' instead of 'carers', and 'the refined SAFER-MH' rather than 'the revised SAFER-MH'.	I have made these changes to the wording.
Methods – again, for consistency is it worth saying 'unpaid carers'? This also applies throughout the article. Either make them carers, or unpaid carers, not a mix of both unless there is a specific reason.	I have added unpaid to any instances throughout the articlce that are currently missing it.
Methods, Participants – I think it should be 'of the team and social media'. It should also be 'NHS Trusts' to match the abstract, not 'NHS trusts'.<br>Where is point 1) for the exclusion	I have made all of these changes.

criteria?	
What does PIS mean? (I know, but you haven't said it in the text)	I have spelt this out in full
I realise it might be due to the journal style, but I'm still struggling with the sub-headings to work out what level things are. For example, what are the sub-sections within 'Procedure'? I assume it's everything up to Data Analysis, but it's not clear. Even just putting the third level headings in italics, or inset slightly would provide some clarity.	I have added italics for the third level subheadings.
Figure 1 – I didn't think I could see the figure initially as it is not labelled, but I am not sure you can 'insert' it in two places.	I have deleted one instance.
Methods, Procedure, 'Think Aloud' Interviews – it would be good if the numbered points were in the style '1)' to match other parts of the article, rather than being '1.'. Also, using 'Think-aloud' does not match the rest of the article.	I have made these changes, thanks for noticing another incorrect think aloud.
Methods, Data analysis – should it be 'emerge organically and be refined' rather than 'emerge organically and we refined'? The sentence 'Two researchers…approach' feels odd as you've already said about the thematic analysis and developing codes inductively, so the sentence is a bit repetitive. If you want to say who carried out the coding, you could combine it with the next sentence to be something like 'After conducting initial coding independently, two researchers (GH, NT) met regularly…'	It should be were refined, I have changed this.<br><br>I have changed the text around two researchers in line with your recommendations.
Methods, Ethical considerations – you need to say what GDPR stands for. Also, you've got a reference for the Mental Capacity Act (see your Discussion section) so you could use it here too.	I have added both
Results – again, I'm struggling with the sub-headings. In this section you've got headings for Participants, Workshops, 'Think Aloud' Interviews, Workshops, 'Think Aloud' Interviews. I assume the	I have added italics for third level subtitles as per your previous recommendations.

first set is within the 'Participants' section, and the first set are their own sections, but it's not clear. Either you need to find a better way to show different levels of sub-headings, or change them to something like Workshop Participants, 'Think Aloud' Interview Participants, Workshop Findings, and 'Think Aloud' Interview Findings so they are self explanatory.	
Results, Workshops – the sentence 'Key workshop findings…accessibility' doesn't really add anything and is more of a statement. I think it could be deleted. Also, it would be good if the numbered points were in the style '1)' to match other parts of the article, rather than being '(1)'. If you are presenting SAFER-Dem prototype 1 (i.e. one prototype even if you had multiple copies of it), should it be 'An initial prototype…was' rather than 'Initial prototypes…were'?	I have deleted that sentence.<br>I have used single brackets as in earlier sections.<br><br>You are correct about changing prototype to singular, I have made this change.
Results, 'Think Aloud' Interviews – it should be 'and the second' instead of 'and second'. I'm not sure the sentence 'However, further implications…care bundle' makes sense. Is there a word missing?	I have deleted the word however, I think this was the problem.
Make it clear that 'Communication' and 'Environmental Issues' are sub-headings under 'Dissatisfaction with current discharge procedures'. I know you say in the text that they are themes within it and Table 1 reinforces it, but the reader should know from the sub-headings too.	I have changed these to italics hopefully that will solve this problem.
The following are all different levels of sub-heading, but it's difficult to tell as they all look the same - Improvements of the SAFER-Dem care bundle, Appropriateness, Usefulness. It also makes it difficult to know when all of the content for one level has been presented, and we're now back at a higher level. It relies on the reader to keep going back to list of themes and sub-themes (or Table	I have also used italics here to

1) to work out where they are, and it shouldn't be like that. It should be clear from the text.	
Consistency – are quotes in "" or '' or nothing, as all are used in different places? Also, are they in italics or not italics as again there is a mix? How is the person being quoted shown, as sometimes you use — and sometimes -? Also, sometimes there is s space after it, and sometimes not, and sometimes they are in bold.	I have changed any " to ' added spaces after each one and removed the erroneous item that was bold.
Resource format – the sentence 'Whilst many of…certain questions' does not make sense. In particular, it's the bit 'participants suggested, recommending'.	I have deleted the word recommending
Emotive responses – saying 'Using the word or 'worries'' is confusing. Have you missed out a word (i.e. using the word 'XXX' or 'worries') or should the 'or' not be there?	I have deleted the word or
People involved – the sentence 'Views were divergent…capabilities' would benefit from a ; after 'participants'. There are other sentences in the article where a ; would make them easier to read, but I'm not going to point out every instance.	I have changed this.
Output of 'Think Aloud' Interviews – please do something with the first sentence as it has three part separated by commas, that don't actually seem to belong together.	I have changed this.
Discussion – RCT? You need to tell your readers what this stands for.	I have spelt this out
Research, Policy and Practice Implications – please revisit the sentence 'Potential barriers…organisational commitment. It either needs a ; or to be split into two sentences.	I've separated this into three sentences and added a few words to improve clarity.
Conclusions – the sentence 'Participants recognise…be necessary' needs work. I can't quite work out what it's trying to say. Also, is it about 'further' refinements being needed, or is it talking about the	I have rewritten this sentence.

refinements that have already been made?	
